# Valorization of Fishmeal Wastewater for Polyhydroxyalkanoate (PHA) Production by *Bacillus cereus*: Process Optimization and Scale-Up

**DOI:** 10.3390/polym18091044

**Published:** 2026-04-25

**Authors:** Zeinab Ehsan-nasab, Ali Taheri, Masoud Dehghani Soufi

**Affiliations:** 1 Department of Fisheries, Faculty of Marine Sciences, Chabahar Maritime University, Chabahar 9971778631, Iran; ehsannasabzeinab@gmail.com; 2 Department of Agrotechnology, College of Abouraihan, University of Tehran, Tehran 1417935840, Iran; dehghaisoufi@ut.ac.ir

**Keywords:** fishmeal wastewater, polyhydroxyalkanoate (PHA), *Bacillus cereus*, bioplastic, 3-hydroxybutyrate-*co*-3-hydroxyvalerate

## Abstract

Recently, polyhydroxyalkanoates (PHAs) have gained significant attention as a bioactive material for replacing petrochemical plastics. PHAs can be produced by microorganisms growing on sludge substrates. In this study, fish-processing wastewater was investigated as an alternative substrate for PHA production using *Bacillus cereus*. Wastewater dilution, carbon-to-nitrogen ratio modification, and the addition of fish oil as a lipidic substrate were examined, and bacterial growth and biopolymer production were optimized. First, wastewater was diluted (25–100%) and examined. The 50% dilution treatment was selected, yielding a CDM of 0.426 g/L and a PHA content of 6.69%. In subsequent steps, the effects of wastewater fermentation and bacterial adaptation prior to the main production processes were investigated. According to the results, the 50% and 100% fermented treatments exhibited higher CDM values (0.970–1.022 g/L) compared to the non-fermented treatments. Cultures inoculated with adapted bacteria showed superior performance (CDM: 1.455 g/L, PHA: 0.499 g/L, PHA content: 34.63%) relative to non-adapted treatments. The effect of the carbon-to-nitrogen (C/N) ratio was also optimized by supplementing two carbon sources: glucose and crude fish oil. The optimal treatment T1 (effluent + 0.6 g/L glucose) had a CDM of 1.32 g/L and a PHA content of 0.215 g/L. Treatment 1, which consisted solely of effluent and fish oil, exhibited higher values (CDM: 1.12 g/L, PHA: 0.65 g/L) and was therefore considered the cost-effective treatment. Subsequently, a scale-up process was conducted in a 4 L bioreactor over 300 h under semi-continuous, long-term cultivation. The optimal harvesting time for the biopolymer was achieved during the fourth cycle (180–240 h). The produced biopolymer was characterized using FTIR, NMR, TGA, DSC, SEM, and XRD analyses, confirming the production of a copolymer, specifically poly(3-hydroxybutyrate-*co*-3-hydroxyvalerate). This study used wastewater from the fish industry for the production of biodegradable polyhydroxyalkanoates.

## 1. Introduction

Researchers and businesses are increasingly working on the development of biodegradable polymers because plastic pollution is becoming a significant problem worldwide [1]. Scientific research has focused on polyhydroxyalkanoates (PHAs), which have garnered considerable attention as biodegradable substitutes. Microorganisms produce PHAs, which are natural polyesters, when nutrients are limited. They have several advantageous features, like being able to be processed thermoplastically, breaking down entirely in soil or marine settings, and being biocompatible [2,3,4].

Therefore, PHAs are the foundation of the circular bioeconomy. The primary impediment to the expansion of commercial production is the expense associated with biomass carbon sources and downstream product recovery [5,6,7]. A pertinent research domain focuses on innovative pathways for sustainable and economical carbon feedstocks to render the expansion of PHA production and utilization financially viable and environmentally friendly. Given these economic constraints, an increasing volume of research has been dedicated to the valorization of economically viable industrial and agricultural waste streams [8,9,10].

Fish-processing wastewater (FPW) is a substrate that has many advantages. To produce FPW in the fishmeal industry, fish are pressed and cooked, and FPW is subsequently separated. FPW contains a large amount of organic matter, as shown by its high chemical oxygen demand (COD) and high levels of nutrients, such as soluble proteins, free amino acids, lipids, and residual sugars [11,12,13,14]. FPW is energy-dense, making it a suitable medium for microbial fermentation. However, it is also difficult to process because it has various components and contains substances that can slow down the process, such as ammonia and fats. Pretreatment may be required before use [7,15]. Using FPW as a carbon source for PHA production replaces conventional carbon sources used in manufacturing, thereby reducing pollution from FPW discharge and supporting the growth of the circular bioeconomy [16].

Recent studies have reported various bacterial genera, notably Bacillus, as essential for the synthesis of polyhydroxyalkanoates (PHAs) from complex waste substrates due to their robustness, varied metabolic pathways, and cultivation simplicity [1,17]. *B. cereus* has attracted significant interest as a proficient PHA producer owing to its ability to use various carbon sources, rapid growth rate, and adaptability to non-sterile environments [18,19,20,21]. Specifically, *B. cereus* has been documented to accumulate PHAs, making up 61.6% of the cell dry weight (CDW) when grown on corn starch residues as the primary carbon source [22]. This species can break down complex substrates, such as fish oil, amino acids, and glycerol, which facilitates the conversion of nutrient-rich waste, such as fish-processing wastewater (FPW), into new, useful biomaterials [16,23,24]. Studies using alternative fishery by-products, including shrimp shell waste and seafood processing residues, have demonstrated that appropriate substrate processing and nutrient optimization can enhance PHA production by improving substrate accessibility and metabolic efficiency [11,25,26]. In addition, it has been reported in the literature that fish oil from fishery and industrial by-products can be successfully used as a carbon source for PHA copolymer production by bacteria. These findings demonstrate the potential to valorize organic waste streams, including those associated with fisheries, for sustainable PHA production [26]. These studies show that Bacillus strains can convert fishing industry waste into PHAs. The carbon-to-nitrogen (C/N) ratio, dilution rate, and addition of co-substrates are important factors that affect how fast microbes grow and how much PHA they produce in their cells. Typically, co-feeding of glucose as an energy source and a lipid-rich source, such as fish oil, is used to augment the quantity of PHA monomers in the microbial biomass [16,27]. This method improves the productivity and quality of biopolymers, which makes it easier to produce copolymers with superior properties, such as better biodegradability and mechanical strength. These properties are important for various applications in materials science and packaging. To achieve a cost-effective scaled-up process, these strategies must be employed in conjunction [28].

This study investigated the use of fishmeal wastewater as a carbon source for polyhydroxyalkanoate production by *B. cereus*. A systematic optimization of essential fermentation parameters was performed, encompassing FPW concentration, carbon-to-nitrogen (C/N) ratio, and the addition of co-substrates glucose and fish oil, to improve both biomass growth and PHA yield. The optimization process, initially conducted in flasks, was later tested in bioreactors using periodic feeding strategies to evaluate process stability and long-term effectiveness. Characterization studies were used to examine the properties of the produced polymer.

## 2. Materials and Methods

### 2.1. Microorganism and Culture Conditions

The *B. cereus* strain (PTCC No: 1948; risk group 1) was obtained from the Iranian Collection of Fungi and Bacteria center. The strain was maintained on nutrient agar slants at 4 °C. Before each experiment, the strain was revived by transferring it to nutrient agar plates and incubating at 37 °C for 18–24 h to obtain isolated colonies. Subsequently, a single isolated colony was placed in a 250 mL Erlenmeyer flask containing 50 mL of sterile nutrient broth. This preculture was shaken at 120 rpm and 37 °C until the OD_600_ was between 4.0 and 4.5, representing the middle to late-exponential phase.

### 2.2. Raw Wastewater Preparation and Physicochemical Analysis

#### 2.2.1. Physicochemical Characterization of Raw Wastewater

Raw fishmeal wastewater was sourced from a fishmeal processing plant in Konarak Industrial Town, Iran, right after it was mechanically pressed. This effluent was placed into sterilized, airtight containers and taken to the lab. The containers were stored at 4 °C until the experiments. We examined the physicochemical properties of raw wastewater to determine whether it would be a suitable environment for bacterial growth and PHA production. We dried the sample at 105 °C to determine the total solids (TS) and burned it at 550 °C to determine the total suspended solids (TSS). We used the AOAC Official Method 984.13 (AOAC, 2019) to determine the total Kjeldahl nitrogen (TKN) [29]. We used ASTM D1426-22 to determine the total ammonia nitrogen (TAN) [30] and the closed reflux–titrimetric method for the examination of water and wastewater (APHA, 2017) to determine the chemical oxygen demand (COD) [31]. A linear correlation model was used to determine the total organic carbon (TOC) (Equation (1)) [32].

COD = (49.2 + 3.00) × TOC(1)

#### 2.2.2. Effect of Dilution on Bacterial Growth and PHA Production

We investigated the possibility of using raw fishmeal waste as a fermentation substrate by examining the effect of dilution on the fermentation process. The raw effluent was diluted to 25%, 50%, 75%, and 100% *v/v* by mixing it with sterile distilled water without prior treatment. We used 500 mL Erlenmeyer flasks for batch fermentation. We placed 20 mL of the *B. cereus* culture that had been sitting overnight into each 250 mL flask. All flasks were shaken at 120 rpm and maintained at 37 °C for 60 h. After fermentation, COD, CDM, and PHA accumulation were measured to determine the optimal organic load.

### 2.3. Pretreatment of Wastewater via Batch Fermentation

We used the optimal FPW dilution of 50%, which had already been determined, to study the effects of anaerobic biological pretreatment. We achieved this by fermenting in an oxygen-free environment. We placed the diluted wastewater in 300 mL glass containers with minimal headspace and maintained it at 37 °C for 72 h, gently stirring at 120 rpm. After incubation, the supernatant from the fermentation process was poured off and used immediately with no further treatment. We examined PHA production at two fermentation levels: 50% and 100% (*v*/*v*). In both cases, 10 mL of *B. cereus* inoculum (as described above) was added to 200 mL of working volume and incubated at 37 °C for 60 h with shaking at 120 rpm. Finally, CDM and PHA contents were compared to those of the untreated effluent.

### 2.4. Bacterial Adaptation to Effluent-Based Media

Before starting the optimization trials, *B. cereus* was adapted to improve its performance on the fermentation substrate. We chose 100% fermented effluent as the adaptation medium because previous research has shown that it works well for producing PHA, as shown by Fourier-transform infrared spectroscopy (FTIR). A rapidly growing culture was transferred to 100 mL of washed effluent (50 mL) and incubated at 37 °C and 120 rpm for 60 h.

### 2.5. Assessing the Impact of Inoculum Adaptation on Bacterial Proliferation and PHA Synthesis

A study was conducted to determine the usefulness of a pre-adapted inoculum. First, 50 mL of an acclimated *B. cereus* stock culture was added to the fermented effluent that had been diluted to 50% or used at full strength (*v*/*v*). The samples were incubated under standard conditions (37 °C, 120 rpm, for 60 h), and the resultant biomass and PHA were directly compared to those from prior experiments that used a non-adapted inoculum from nutrient broth.

### 2.6. Optimization of the Carbon-to-Nitrogen Ratio

The C/N ratios of 1:1, 10:1, and 100:1 were produced by adding glucose to 100% pre-fermented wastewater, and the effect of the carbon content was measured. The experimental design included a negative control (unsupplemented wastewater) and a positive control (synthetic nutrient medium). All cultures were fermented at 37 °C for 60 h with stirring at 120 rpm. After fermentation, we examined the CDM and PHA levels to determine how the amount of carbon affected bacterial development.

### 2.7. Impact of Raw Fish Oil on PHA Synthesis

Synthetic nutritional medium and secondary effluent with glucose were used as the optimal pretreatment tests for boosting PHA production. This study used four different treatments: (i) fish oil; (ii) a combination of fish oil and glucose; (iii) fish oil with synthetic nutrients; and (iv) a mixture of fish oil, glucose, and synthetic nutrients. Controlled conditions of 37 °C and 120 rpm were employed for the fermentation of all treatments, and biomass and PHA yields were investigated.

### 2.8. Monitoring Fermentation and Scale-Up

Following the outcomes of preceding stages, a scale-up fermentation was performed in a 4 L bioreactor for over 300 h using a mixture comprising 100% fermented wastewater (2000 mL, 60%), seed effluent (1200 mL, 35%) containing adapted bacteria, and fish oil (180 mL, 5%). The temperature was maintained constant at 37 °C, and agitation was set at 120 rpm. Dissolved oxygen (DO) and pH were recorded but not regulated under previously reported protocols for bioreactor-scale PHA production [33]. To sustain microbial activity, a 20% medium refresh was performed every 60 h as a repeated-batch strategy. Process performance was monitored regularly by measuring cell dry mass (CDM), PHA content, pH, DO, and chemical oxygen demand (COD), enabling a long-term kinetic analysis of growth, substrate consumption, and PHA synthesis.

### 2.9. Methods for Analysis

#### 2.9.1. Extraction and Measurement of PHA

Sodium hypochlorite was used for PHA extraction. To shatter the cells, sodium hypochlorite was first used on dried cell material. Then, the polymer was collected by centrifuging. Then, the solid was rinsed with distilled water, methanol, and acetone. After that, it was dissolved in chloroform, and the solvent was removed, and the PHA was gathered. The PHA weight was determined by weight and represented as a fraction of the dry cell mass, in accordance with prior research (Equation (2)) [34].

(2) $$\mathrm{PHA}\;\mathrm{content}\;(\%)=\frac{Weight\;of\;PHA\;(g)}{Weight\;of\;dry\;biomass\;(g)}\;\times 100$$

#### 2.9.2. Fourier-Transform Infrared Spectroscopy (FTIR)

Fourier-transform infrared (FTIR) (PerkinElmer, Waltham, MA, USA) was used to examine the chemical composition of the extracted PHA. The ATR mode in the wave-number range of 4000–400 cm^−1^ was used. Different scans were performed for each sample in order to guarantee uniform results [34].

#### 2.9.3. Nuclear Magnetic Resonance (NMR) Spectroscopy

A thorough structural analysis of the purified PHA was performed using nuclear magnetic resonance (NMR) spectroscopy. For this purpose, samples were prepared by dissolving 10–20 mg of the polymer in deutero-chloroform (CDCl_3_). The ^1^H NMR spectra were obtained at ambient temperature using a 400 MHz spectrometer (Bruker, Berlin, Germany), with CDCl_3_ serving as the internal standard [35].

#### 2.9.4. Differential Scanning Calorimetry (DSC)

We used a PerkinElmer DSC 8000 (Waltham, MA, USA) to test the thermal properties of the PHA. A hermetically sealed aluminum pan containing approximately 5–10 mg of dried polymer was subjected to a heat–cool–heat cycle that ranged from −50 °C to 200 °C at a rate of 10 °C/min under a nitrogen purge. We used the second heating cycle to determine the values of T_m_, Tg, and Xc. This was done to ensure that the data accurately showed the natural properties of the material with no processing history [36,37].

#### 2.9.5. Thermogravimetric Analysis (TGA)

A PerkinElmer TGA 4000 (Waltham, MA, USA) instrument used for thermogravimetric analysis (TGA). A sample of dried polymer weighing approximately 5–10 mg was heated in a nitrogen atmosphere (20 mL/min) from ambient temperature to 600 °C at a rate of 10 °C/min. The onset degradation temperature (T_onet_), the temperature corresponding to the maximum degradation rate (T_max_), and the residual mass were determined.

#### 2.9.6. Scanning Electron Microscopy (SEM)

Morphology was studied using scanning electron microscopy (SEM). Biomass with PHA was deposited on aluminum stubs and coated with gold using a Quorum Q150R ES sputter coater (Quorum Technologies, East Grinstead, UK) to prepare the sample. Images were captured using a TESCAN MIRA3 (Brno, Czech Republic) at a voltage of 10 kV.

#### 2.9.7. X-Ray Analysis

X-ray diffraction (XRD) analysis for the extracted polymer (PHA) was performed using a PANalytical X’Pert PRO diffractometer (Malvern, UK), which employed Cu Kα radiation (λ = 1.5406 Å) at 40 kV and 30 mA. The scans were conducted over a range of 5° to 60° (2θ), with a step increment of 0.02° and a scanning speed of 2°/min [38]. The peak areas were calculated and used to measure crystallinity (Equation (3)).

X% = (∑Ap/A_total_) × 100 (3)

where ∑Ap is the sum of peak areas, and A_total_ is the total area of the spectrum.

The Origin Pro software (update 2025b, 10.25) was used for this analysis.

### 2.10. Statistical Analysis

All quantitative measurements were conducted in triplicate. To verify the assumption of normality, the Shapiro–Wilk test was applied to the dataset. Group comparisons were performed using a one-way analysis of variance (ANOVA), followed by Tukey’s post hoc test for the identification of homogeneous subsets. Statistical significance was defined as a *p*-value ≤ 0.05. The results are reported as mean values accompanied by their standard deviations.

## 3. Results and Discussion

### 3.1. Physicochemical Characterization of Fishmeal Wastewater

Physicochemical characterization proved that FPW raw fishmeal wastewater is a nutrient-rich substrate and is suitable for use as a source for microbial PHA production, as shown in Table 1.

The pH was slightly acidic, and the chemical oxygen demand (COD) and total organic carbon (TOC) showed a high concentration of solids and organic matter in water. In addition, FPW had high TS and TSS values of 144,533 mg/L and 80,244 mg/L, respectively, showing a high content of undissolved organic matter. As expected, the nitrogen content was high. Characterization showed a highly reduced environment, which, although nutrient-dense, is most likely inhibitory to microbial growth without treatment. This is consistent with most seafood processing wastes [39,40]; therefore, diluting and acclimating the substrate for microbial use is an important first step.

### 3.2. Effect of Effluent Dilution on Biomass Growth and PHA Production

To assess the impact of substrate concentration on *B. cereus* growth and PHA accumulation, fishmeal wastewater was diluted to 25%, 50%, 75%, and 100% using sterile distilled water and allowed to sit for 60 h. These treatments resulted in notable differences in cell dry mass (CDM), PHA content, and total PHA yield (*p* < 0.05) (Table 2).

The highest CDM was found at 75% dilution (0.726 ± 0.051 g/L), but the highest PHA content was in the 25% dilution treatment (8.87 ± 3.45%). There were some differences, but the PHA yield did not change significantly between the treatments. The maximum PHA yield was observed at 75% dilution (0.052 ± 0.02 g/L). At 75% dilution, increased substrate concentration resulted in greater biomass production; however, the PHA generated at this level showed unclear FTIR spectra, suggesting lower structural quality. At 100% (undiluted wastewater), PHA levels decreased dramatically, likely due to nitrogenous toxicants and inhibitory chemicals, such as ammonia, as previously observed in high-strength organic waste streams [41,42,43]. This corroborates previous research that emphasized the importance of carbon availability for PHA production [44,45,46].

The PHA yield did not show any differences among the treatments. Therefore, quantitative criteria alone are not enough to determine the optimal conditions, and a qualitative description of the polymer is necessary. FTIR analysis showed that the PHA generated at 50% dilution exhibited commendable structural integrity. This finding is in line with earlier studies that showed how the composition of the substrate and the amount of organic matter affect the polymer’s purity and structural integrity [44,47]. A 50% dilution was selected for the subsequent phase of the study.

### 3.3. Effect of Fermented Substrate on Biomass Growth, PHA Production, and Polymer Quality

The results show that the use of fermented substrates is essential for enhancing both cellular proliferation and PHA synthesis. Higher CDM values (0.970–1.022 g/L) were observed in the 50% and 100% fermentation treatments compared to non-fermented treatments (*p* < 0.05) (Table 3).

This enhancement in growth is consistent with previous studies, which suggest that fermentation improves substrate bioavailability by decomposing complex organic materials, reducing inhibitors, and generating volatile fatty acids (VFAs), which serve as preferred metabolic precursors [48,49].

The fermented treatments also produced more PHA than the non-fermented treatments. The PHA content in the 50% and 100% fermented treatments was 0.375 g/L and 0.249 g/L, respectively. The amount of PHA in the biomass also increased, from 31.86% in the 50% fermented treatment to 25.64% in the 100% fermented treatment. The slightly lower PHA percentage in the 100% treatment could be due to the higher organic load and the resulting change in how the body uses its energy when there is more substrate present. This has been linked to carbon stress and osmotic imbalance [50,51].

The 100% fermented method had less PHA by weight and concentration than the 50% fermented treatment, but the quality of the extracted polymer was much better. The FTIR spectra showed that the polymer chains were arranged in a more orderly way. This could indicate that the crystallinity is higher or that the monomer composition is different. When fermentation is completed, the VFA profile usually becomes more diverse. This can change the biosynthetic pathway, thus producing polymers with more uniform or structurally ordered properties, or even copolymers, which exhibit stronger and more distinct FTIR signals [52,53,54]. The 100% fermented treatment produced less PHA than the 50% fermented treatment, but the polymer structure and spectral quality were better. Based on these results, the 100% fermented treatment contained a high amount of PHA and was better in terms of chemical purity and structural quality. It is scientifically sound to choose the 100% fermented treatment for further research because the structure, crystallinity, and monomer composition of PHAs have a significant effect on their performance and usefulness in advanced materials [53].

### 3.4. How Bacterial Adaptation Affects the Growth of Biomass and PHA

**Figure 1:**
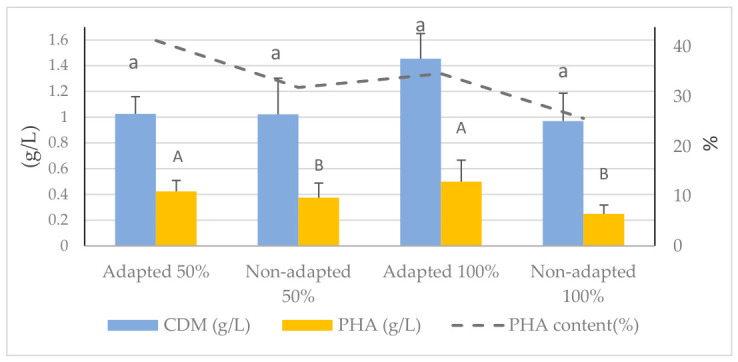
Comparison of cell dry mass (CDM) and PHA production in adapted and non-adapted *B. cereus* cultures at 50% and 100% fermented fishmeal effluent. Different letters show significant differences in each treatment (*p* < 0.05).

The process worked much better when an adapted *B. cereus* inoculum in 100% fermented effluent was used. The final cell dry mass was 1.455 ± 0.195 g/L, the PHA concentration was 0.499 ± 0.167 g/L, and the PHA content was 34.627 ± 11.19%. This means that there was 25.59 ± 3.165% PHA in the cells. This was a big step up from the non-adapted system, and it demonstrated that the pre-adaptation method enhanced the ability of the bacteria to break down the complex effluent (Figure 1).

The results show that microbial pre-adaptation is necessary for increasing biomass growth and PHA production when fermented effluents are used as the carbon source. Figure 1 shows that for all the parameters examined, the adapted cultures performed better than the non-adapted treatments. Adaptations made growth much more efficient. The adapted 100% treatment produced the highest cell dry weight (1.455 g/L) and total PHA accumulation (0.499 g/L). This improvement is likely due to the physiological stress response that occurs during the adaptation phase. This response leads to overexpression of genes that help move and break down complex organic substrates found in the fermented effluent [54]. The modified 50% treatment had the highest amount of PHA inside the cells (41.3%); therefore, carbon was turned into storage polymers quickly, even though the solution was slightly diluted.

Adaptation changes the structure of the polymer and its quantity. Fourier-transform infrared (FTIR) analysis showed that the PHA generated by the adapted 100% treatment displayed more distinct absorption peaks compared to the other treatments, implying the development of a more homogeneous and structurally consistent polymer. The increasing level of crystallinity may be due to an increase in PHA synthase activity in pre-stressed cells, leading to reduced impurities [55].

These results collectively highlight the significant influence of microbial adaptability on improving both the output and quality of PHA when employing concentrated, fermented effluents. The adapted 100% culture exhibited superior overall performance across all treatments and was chosen for the next phase of this study.

### 3.5. Glucose Effects on PHA Production

**Figure 2:**
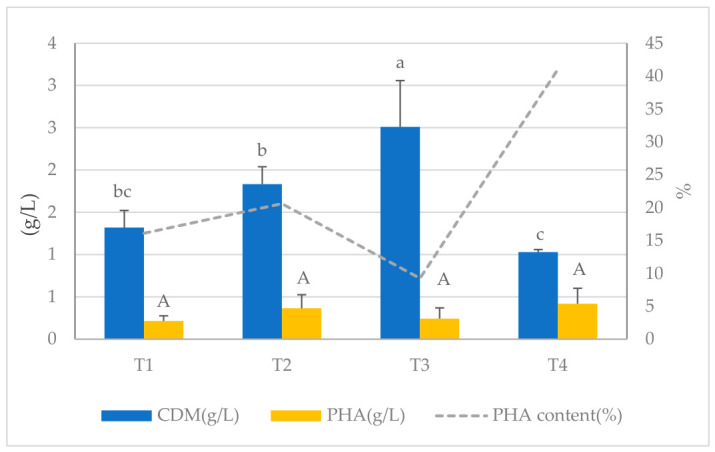
Comparison of cell dry mass (CDM), PHA yield, and PHA content (% of glucose supplementation). Different letters show significant differences in each treatment (*p* < 0.05).

The control treatment (100% effluent without glucose) had a CDM, PHA concentration, and PHA content of 1.455 g/L, 0.499 g/L, and 34.62%, respectively. In Treatment 1 (effluent + 0.6 g/L glucose), the CDM (1.32 g/L) and PHA (0.215 g/L) levels were only slightly lower than those of the control (Figure 2).

The negligible effect of glucose addition is likely due to the elevated organic matter concentration in the effluent, which promotes rapid glucose utilization and plays a limited role during the PHA accumulation phase [13,56]. This treatment also showed PHA peaks in FTIR measurements, meaning the polymer was still being produced. The CDM level in Treatment 2 (effluent + 6 g/L glucose) increased to 1.833 g/L, but the PHA generation (0.365 g/L) and PHA content (20.59%) decreased even further. The increase in biomass formation is beneficial, but the drop in the PHA fraction shows that carbon is being diverted to produce extracellular polymeric substances (EPSs), which are mostly made up of extracellular polysaccharides. When there is a moderate amount of glucose available, cells can produce more EPS. This helps them store extra carbon and energy and makes them more resistant to environmental stress [13,57]. EPS are produced from glucose and other monosaccharides, and their production competes with the PHA pathways. Because of this, a higher C/N ratio encourages the production of EPS over PHA [13]. The FTIR test of this treatment showed peaks that were noisy and uneven. This supported the idea that there were unwanted side products and that the polymer was less pure [58].

In Treatment 3 (effluent + 60 g/L glucose), the cell dry mass (CDM) achieved its peak value of 2.51 g/L; however, both the PHA concentration (0.242 g/L) and PHA content (9.26%) exhibited a significant decline in value. This decrease is due to catabolite repression and the fact that carbon flux is more likely to be directed toward biomass production than polymer storage. Increased glucose levels lead to higher glycolytic flux, which stops β-oxidation and lowers the amount of acetyl-CoA available for PHA synthesis [56,58]. These results align with prior research, demonstrating that excessive glucose supplementation in a fatty-acid-rich environment reduces PHA productivity in multiple species, including *Cupriavidus necator* and *Bacillus megaterium* [56,57]. A similar pattern has been noted in lipid-rich wastewater, where low glucose levels promote initial development, whereas elevated amounts impede fatty acid use [13]. In Treatment 4 (effluent + synthetic mineral salts), the CDM was 1.03 g/L, and the PHA concentration was 0.419 g/L. This means that this treatment had the highest PHA content (41.03%) of all the treatments. This enhancement is due to a better balance of nutrients, especially the optimization of the carbon-to-nitrogen ratio and increased availability of cofactors that are important for energy metabolism [58]. The practical advantages of this treatment are still restricted.

Cultivation of *B. cereus* in raw effluent from a fishmeal production facility revealed that this wastewater has a significant inherent ability to meet the nutritional and metabolic needs of the microorganism, facilitating both growth and PHA synthesis without requiring external carbon supplementation. These findings confirm that the organic constituents present in the effluent can efficiently enter energy-generating pathways and provide the acetyl-CoA required for PHA biosynthesis [13,56,58].

### 3.6. The Impact of Raw Fish Oil Supplementation on PHA Production

**Figure 3:**
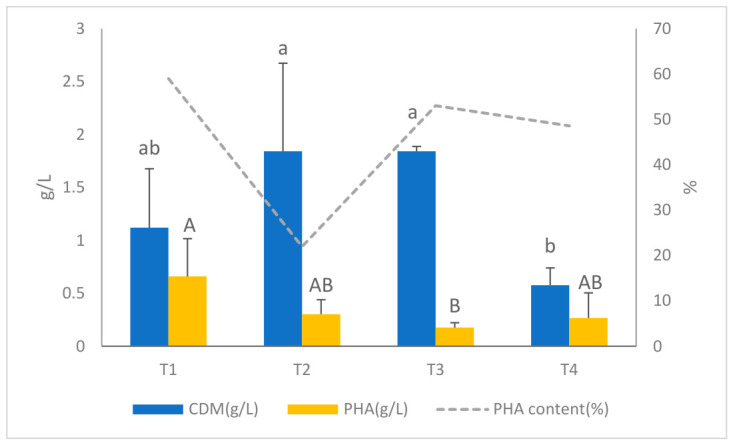
Comparison of cell dry mass (CDM), PHA yield, and PHA content (%) of raw fish oil supplementation. Different letters show significant differences in each treatment (*p* < 0.05).

When crude fish oil was added to the mix, *B. cereus* produced significantly more PHA. This suggests that it might be a good source of carbon for growing microbes in waste-derived media. Four treatments were tested to determine how they affected biomass formation, PHA accumulation, and polymer quality. These treatments included fermented and inoculated effluents that were high in fish oil content. Treatment 1, which used only effluent and fish oil, was the most effective of all the treatments, as shown in Figure 3. This treatment produced a cell dry mass (CDM) of 1.12 g/L and a PHA concentration of 0.65 g/L, resulting in a PHA content of almost 59%, which was the highest among all treatments.

FTIR analysis confirmed the polymer’s quality by showing characteristic absorption bands and a clear carbonyl peak at about 1730 cm^−1^, indicating strong polymer accumulation [26,53]. In contrast, treatments with glucose (T2), synthetic medium (T3), or a mix of the two (T4) showed significantly less PHA accumulation (0.30, 0.18, and 0.27 g/L, respectively) and significantly less PHA content (21.9%, 53.0%, and 48.5%, respectively) than the control. T2 and T3 showed small increases in biomass because they had extra carbon or minerals. The metabolic flow switched from the pathways that produce PHA to growth-associated pathways that do not produce polymers. This pattern aligns with previous studies showing that monosaccharides, including glucose, can augment glycolytic flux, consequently redirecting acetyl-CoA from PHA synthesis [59]. Similar results have been noted in various Bacillus species, where the introduction of glucose often promotes growth while preventing polymer accumulation due to carbon overflow metabolism [51].

Fish oil is a good source of carbon because it contains medium- to long-chain fatty acids (C14–C22), which are used directly in the synthesis of PHA through β-oxidation and other lipid-dependent metabolic pathways [27,53]. The slow release of fatty acids from emulsified oil in the medium helps keep the carbon levels stable. This helps cells maintain metabolic activity and facilitates the conversion of carbon into polymers. Empirical evidence shows that lipid-based substrates more effectively initiate β-oxidation and promote the accumulation of PHA and copolymers with superior material properties compared to carbohydrate-based substrates [60,61].

Zmila et al. [26] showed that the different fatty acids in fish oil may help different monomer units fit together better, enhancing polymer flexibility and expanding its potential applications. Adding lipids to the diet may also change the redox balance inside cells, especially the ratios of NADH/NAD^+^ and NADPH. These ratios are very important for the pathways that produce polymers that are reductive. Research indicates that lipid metabolism establishes a redox environment more conducive to PHA accumulation than sugar catabolism [51,59], although the precise regulatory mechanisms remain unclear.

Overall, the current findings show that crude fish oil serves as a highly effective and cost-efficient carbon source in effluent-based culture systems, resulting in enhanced PHA synthesis and improved polymer quality compared to glucose and synthetic nutrient additions. These findings align with current research that emphasizes the effectiveness of fatty substrates and fish-processing by-products as sustainable feedstocks for ecologically responsible PHA production [27,61].

### 3.7. Scale-Up Performance of Bacillus cereus in Repeated-Batch Fermentation

**Figure 4:**
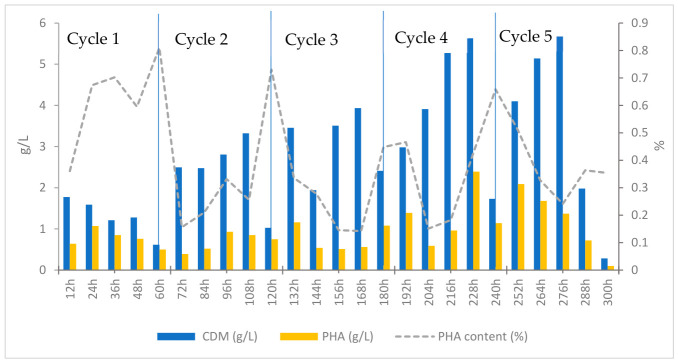
The patterns of CDM changes, PHA content, and intracellular accumulation percentage in the 300 h scale-up cultivation of *B. cereus* in a 4 L bioreactor.

**Figure 5:**
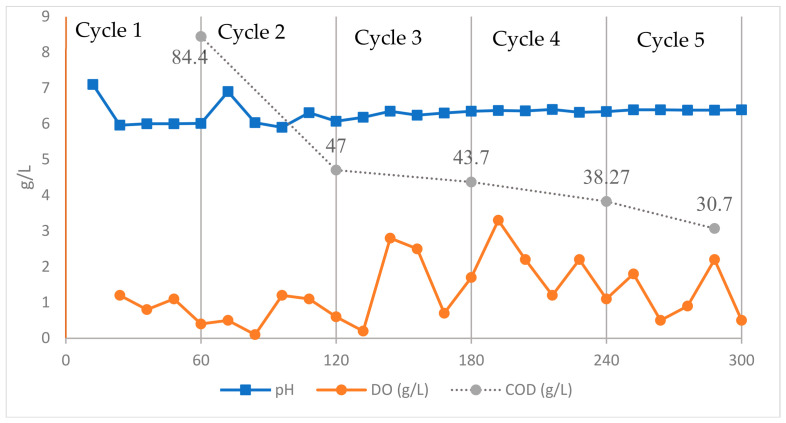
COD, DO, and pH behavior during the scale-up of 300 h cultivation of *Bacillus cereus* in a 4 L bioreactor.

The 300 h cultivation of *B. cereus* in a 4 L bioreactor using the “fish wastewater + fish oil” treatment showed a multiphase dynamic process that was significantly influenced by the substrate quality, rheological state of the culture, and oxygen limitation. The observed patterns of CDM changes, PHA content, and intracellular accumulation percentage (Figure 4), as well as COD, DO, and pH behavior (Figure 5), revealed that the system evolved from a stable growth–storage phase during the initial cycles to a limiting, intermittent, and ultimately deteriorating condition in the later cycles.

During the first cycle (0–60 h), *B. cereus* showed a metabolic profile that was in line with its usual activity in lipid-rich environments. This is called the “metabolic activation phase.” The data from 12 to 60 h show that the bacteria used a significant amount of substrates and activated lipid-catabolic pathways. The COD level dropped from 84,400 mg/L at 60 h to 47,033 mg/L at 120 h. This aligns with the documented fast degradation of fish-processing effluent by microorganisms. Similar initial COD depletions have been observed in fermentations employing fish waste and fish-oil-derived effluents [62]. The substantial decline in dissolved oxygen, decreasing from 1.1 ppm after 12 h to around 0.4 ppm at 60 h, corresponds with the recognized elevated oxygen requirement of lipid β-oxidation and oxidative fatty acid metabolism compared to carbohydrate utilization [59]. This DO pattern during fermentation has been documented in fish oil, vegetable oil, and long-chain fatty acids.

Simultaneously, the biomass and polymer levels at the end of the cycle were recorded as CDM ≈ 0.61 g/L, PHA ≈ 0.50 g/L, and intracellular content ≈ 81%. The increase in biomass and polymer levels during this period suggests that PHA accumulation was largely associated with cell growth while the substrate was actively consumed. Similar levels of intracellular PHA accumulation have been reported for *Bacillus megaterium* and other *Bacillus* species cultivated on fatty acid-rich substrates [54,57]. The pH decreased from 7.1 to 6 and decreased at the start of each cycle. This may be due to the breakdown of fatty acids, which makes the environment acidic. As the fermentation procedure is considered a complex phenomenon, other reasons, including CO_2_ production, accumulation of organic acids, and protein hydrolysis, should also be considered. The pH remained between 6.0 and 6.4 in the subsequent cycles, which shows that the metabolic activity had changed. Similar pH patterns have been observed in the fermentation of protein–lipid waste streams. The patterns of quick COD decrease, steady DO depletion, and concomitant rises in CDM and PHA are all in line with the “metabolic activation phase,” which is common in semi-continuous lipid fermentations. This is when fatty acids are consumed the most, and PHA storage begins [42,51,63].

After the initial 20% medium refresh after 60 h, the system went into a more balanced metabolic phase because the biomass and nutrients were spread out more evenly. In the second cycle, *B. cereus* kept growing at a steady pace and got better at storing polymers. The intracellular content was approximately 30–31% because the quantity of CDM increased from approximately 3.3 g/L to 4.6 g/L, and the amount of PHA increased from 0.85 g/L to approximately 1.32 g/L. This modest rise in polymer building suggests that the carbon flow stayed concentrated on storage metabolism, even when the growth rate slowed down. The amount of COD removed decreased from 395 mg/L to approximately 365 mg/L. This decline was caused by the utilization of lipid fractions that break down slowly and proteins that are only partially broken down. These become more important after the initial, easily usable substrates are depleted. Lipid-rich effluents have also been shown to behave in the same way, with COD removal becoming less effective with each feeding cycle [62]. Dissolved oxygen (DO) remained low during Cycle 2 and only increased for a short time after the medium was added again. This pattern shows that oxidative activity is continuous and that fatty acid β-oxidation requires more oxygen than carbohydrate degradation. The carbon flux can shift from biomass synthesis to intracellular storage polymers in response to microaerobic conditions that cause nutrient imbalance [54,57].

In the pH range of 6.0–6.4, enzymatic activity is high and reduces the inhibition caused by acidification. From a physiological perspective, prolonged exposure to lipid substrates likely enhances sequential β-oxidation and NADPH-generating pathways, modifying the intracellular redox equilibrium to promote PHA production. This metabolic change explains why the CDM increased slightly and why polymer accumulation increased more than in Cycle 1. This is in agreement with previous studies on fermentations involving fish oil. Cultures exposed to prolonged feeding exhibited reduced growth rates and a gradual increase in intracellular PHA accumulation. Longer feeding times caused cultures to grow more slowly and store more PHA inside their cells, which shows that they were storing more polymers. The current study [54,57,64,65] exhibited a similar metabolic pattern characterized by moderate biomass growth and improved carbon-to-polymer conversion efficiency. Cycle 2 was a time of metabolic change when the culture maintained efficient PHA accumulation even though growth was slower and the COD removal rate was lower. Biomass formation slowed down compared to Cycle 1, but carbon conversion efficiency and polymer storage improved. This is a normal reaction in semi-continuous lipid-rich fermentations [66].

Cycle 3 changed the course of fermentation. The system changed from having significant amounts of soluble substrates to one with little oxygen and long-chain fatty acids (LCFAs). After changing the medium at 120 h, the biomass swiftly increased from around 1.0 g/L to over 3.4 g/L in 12 h. This means that the soluble fatty acids were quickly taken up. However, this increase did not last long, and by 180 h, the CDM had dropped to approximately 2.4 g/L. In semi-continuous lipid-fed bioreactors, soluble fatty acids are rapidly taken up after the medium is renewed but are used shortly thereafter [51,67]. The CDM fell to about 2.4 g/L after 180 h, so cells had to rely on triglycerides and LCFAs that break down slowly after the conveniently available fraction was used up [51,57,65]. These substrates slowed down transport and lowered the total biomass output [51,65]. The polymer content dropped significantly to about 14–15% in the middle of the cycle, when the DO values were at their lowest. This alignment suggests that limiting oxygen is a key factor in changing the flow of carbon from PHA production to keep cells healthy. When there is insufficient oxygen, NADH oxidation slows down, which causes the cell to break down stored PHA to restore redox balance to normal. This is a known stress response in Gram-positive PHA producers [19,68]. Interestingly, the system regained its ability to collect polymers toward the end of the cycle, and the PHA content increased again to approximately 44% [51,67]. This rebound is in accordance with a small rise in DO. This suggests that the cells reverted to a metabolism that generates NADPH for PHA synthesis after breaking down the most recalcitrant LCFAs and lowering the need for oxygen for a short time [63,65]. Microorganisms producing PHA can be grown in emulsified fish oil or grease-trap waste. These species have also been shown to undergo similar “recovery phases” after LCFA-induced stress [19,65].

After changing the medium at 180 h (Cycle 4), the culture recovered its metabolism and stored carbon more efficiently. The environment shifted during Cycle 4 to regulate cellular activity and reactivate the critical biochemical pathways involved in PHA generation after the intense oscillations observed in Cycle 3. At the beginning of this cycle (180 h), the CDM level was about 2.4 g/L. In the next 36 to 48 h, CDM rose sharply, reaching about 5.2 g/L at 216 h and 5.6 g/L at 228 h. This biomass regeneration suggests that portions of the emulsified lipid layer and semi-hydrolyzed proteins, which were not properly metabolized in Cycle 3, became re-accessible during this interval [27,69]. Previous studies have demonstrated that after transient metabolic instability, cells can restart growth by reactivating fatty acid oxidation pathways and lipase activity, restoring growth and polyhydroxyalkanoate (PHA) production [70,71].

The pattern of PHA accumulation also reflects the entry of the culture into a more efficient metabolic state. Although PHA (1.08 g/L at 180 h) showed a slight initial decline, consistent with temporary DO drops and oxygen limitation, it subsequently increased sharply, reaching 2.39 g/L by 228 h. The intracellular polymer content increased to approximately 42–44%. Notably, this increase occurred under persistently low dissolved oxygen (DO) levels. A sharp decline in DO following the activation of β-oxidation indicates a substantial increase in oxygen demand. Under these conditions, the rate of oxygen uptake by cells surpasses the rate of oxygen transfer into the medium. As a result, the carbon flux shifts from pathways that support growth to pathways that store carbon, leading to the accumulation of more PHA [43,58]. Previous studies have validated that “mild oxygen limitation zones” significantly promote PHA production in substrates abundant in fatty acids [27,51,65,69]. The COD profile during this cycle exhibited a continuous decrease, reflecting the consumption of medium-chain fatty acids and hydrolyzed triglyceride compounds that remained unutilized during Cycle 3 owing to oxygen-transfer limitations and reduced hydrolase activity. In Cycle 4, however, the cells entered a phase in which the remaining COD was metabolized with higher efficiency, a pattern frequently reported in semi-continuous and fed-batch lipid-based bioreactors after passing the initial instability window [72,73]. Notably, even when CDM increased in the middle of the cycle, PHA kept increasing instead of declining. This means that the balance of energy inside the cells changed to favor polymer synthesis, and more of the acetyl-CoA generated by β-oxidation was used to produce PHA. This event transpires when the NADH/NAD^+^ and NADPH/NADP^+^ ratios reach thresholds that promote energy conservation and anabolic polymer synthesis pathways over cellular proliferation [43,51].

At the end of the fourth cycle (240 h), the CDM dropped to 1.73 g/L, whereas the PHA content increased to approximately 65%. The decrease in biomass and increase in the polymer percentage indicate that the cells switched to a mode of low growth and high storage. This situation leads to metabolic stress in some cells, while other active cells accumulate PHA quickly, leading to higher intracellular polymer fractions. Therefore, in Cycle 4, storage activity was enhanced, as this was the recovery phase. This observation is in agreement with previously reported metabolic shifts observed in lipid-rich and semi-continuous systems, where, following an initial fluctuation phase, the culture transitions into a performance-optimized stage characterized by peak PHA accumulation [57,64,74].

Following the final medium alteration at 240 h (Cycle 5), the culture transitioned into a terminal phase characterized by metabolic weariness and a reduction in biosynthetic activity. The CDM declined to about 1.7 g/L after 240 h due to dilution following medium replacement. However, the biomass increased again during the first stage to the middle of Cycle 5, increasing from 4.1 to 5.6 g/L between 252 and 276 h of operation. This brief recovery shows that some of the remaining lipid droplets and semi-hydrolyzed proteins were still being broken down.

There are also some reports available on transient metabolic responses in *Bacillus* sp. during the late stages of cultivation [57,74]. The PHA profiles exhibited similar trends. The polymer level increased initially, reaching around 2.09 g/L at 252 h and approximately 50% of the intracellular contents. However, it declined at later times, especially after 276 h.

The PHA content dropped significantly toward the end of the cycle (300 h), even when the COD levels kept declining. This trend strongly suggests that cells were shifting from polymer production to polymer degradation for maintenance under declining oxygen levels and substrate quality. Similar internal polymer consumption under carbon or oxygen limitation has been documented in Gram-positive and other PHA-producing bacteria [51,57,74].

The dissolved oxygen measurements support this view. Despite occasional increases immediately following medium refreshment, DO sharply declined at each subsequent sampling point, remaining at critically low levels throughout most of the cycle. The recurrent imbalance between oxygen demand and oxygen transfer creates a metabolic state in which the β-oxidation pathway is active but is insufficiently supported by the oxygen supply. Under these conditions, oxidative stress and redox imbalance accumulate, ultimately suppressing polymer synthesis and triggering PHA mobilization [51,75,76,77]. COD patterns also showed a decline in culture performance. During the later phases of this cycle, the COD continued to decrease from the 240 h levels to the 288 h levels. However, the COD drop was far smaller than it had been in the previous round. This is typical of systems where the remaining organic load is made up mostly of long-chain fatty acids that are difficult to break down, triglycerides that take a long time to break down, and proteinaceous aggregates that are harder to break down [78,79]. As oxygen becomes less available, its breakdown becomes less effective, which places even more stress on the cells’ metabolism. The dip in CDM observed near the conclusion of the cycle (from 288 to 300 h) was due to the combined effects of substrate exhaustion, low DO, and the high energy demands of maintenance metabolism. The decline in CDM was due to cell lysis and biomass loss as PHA levels fell and cells went through deeper stages of hunger and oxidative stress [51,75]. This change to a “terminal metabolic state” has also been observed in Gram-positive PHA-producing bacteria, which rely heavily on β-oxidation and NADPH-dependent pathways for growth and polymer synthesis [51,57]. In short, Cycle 4 was the optimal time for performance, and Cycle 5 was the start of a decline in metabolic activity. This meant that the culture had reached the point where it could no longer work well under the current conditions.

### 3.8. Analytical Characterization of Extracted PHA

#### 3.8.1. FTIR Characterization of PHA During Scale-Up Fermentation

**Figure 6:**
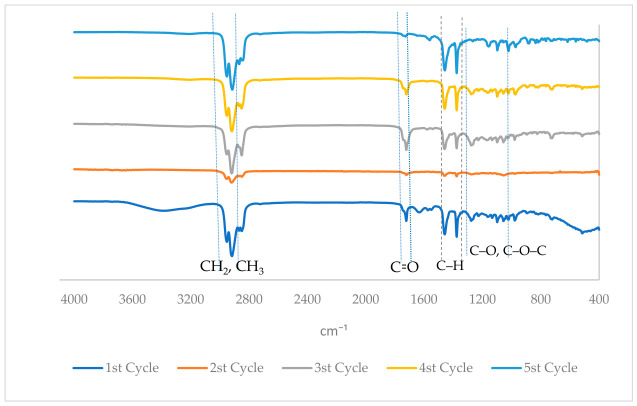
FTIR spectra of PHA samples extracted from *Bacillus cereus* at different fermentation cycles (60 h, 120 h, 180 h, and 240 h).

The spectra from all sampling areas showed typical absorption bands for PHA, which indicated that the polymer produced during scale-up kept its basic chemical structure (Figure 6). The strongest ester carbonyl (C=O) stretching band at about 1720–1735 cm^−1^ was the most noticeable feature of all the spectra. This band is well-known as the main peak of polyesters of the PHA type. The persistence and increase in this band throughout all operational phases indicate that polyester synthesis continued during scale-up [80,81]. There were also a few absorption bands between 2850 and 2950 cm^−1^. The symmetric and asymmetric stretching vibrations of the aliphatic –CH_2_ and –CH_3_ groups, respectively, caused these bands. These bands indicate the presence of long hydrocarbon chains derived from fatty-acid-based substrates. This aligns with the observation that fish oil and lipid-rich effluent are important sources of carbon. Researchers have discovered that PHAs derived from lipid substrates and oily waste streams display analogous spectral characteristics [80,82]. The range of 1450 to 1380 cm^−1^, which is related to –CH bending vibrations, did not change. This means that the structure of the polymer’s alkyl backbone showed little change. The absorption bands in the 1050–1300 cm^−1^ range, which are related to the stretching of C–O and C–O–C ester bonds, show that the polyester backbone remained stable during scale-up [80,83].

A comparison of the spectra from the beginning, middle, and end of fermentation showed that the peaks were not in the same place and were of different intensities. Samples that had significant amounts of intracellular PHA had stronger carbonyl and alkyl stretching bands. This observation suggests that the amount of polymer increased rather than a change in polymer chemistry. This supports the gravimetric and intracellular PHA data collected during the study. Time-course studies of PHA biosynthesis have also found a connection between the intensity of FTIR peaks and the amount of PHA present [80,82]. FTIR analysis showed that the scale-up process did not change or break down the chemicals in the produced PHA. The polymer structure stayed the same during the fermentation process, even when the dissolved oxygen levels, COD, and biomass changed. These results show that *B. cereus* is an effective PHA generator in semi-continuous, lipid-rich, and scaled-up environments. This approach is beneficial for sustained biopolymer production without lowering the quality of the materials.

#### 3.8.2. Nuclear Magnetic Resonance (NMR) Spectroscopy

We used ^1^H nuclear magnetic resonance (NMR) spectroscopy to confirm the chemical structure of the produced polymer. The resulting spectra showed the classic fingerprint of a PHA, specifically matching the poly(3-hydroxybutyrate) or PHB type.

**Figure 7:**
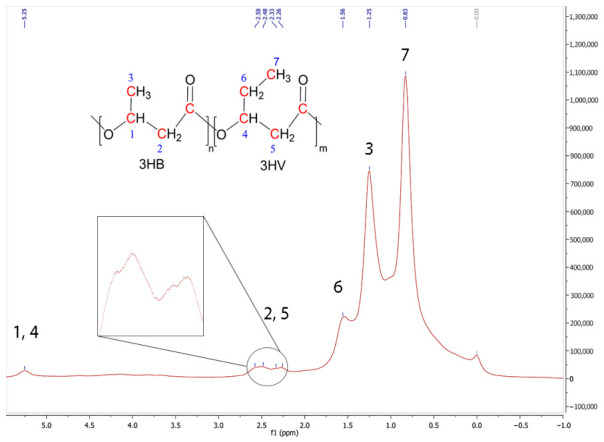
^1^H NMR spectra of extracted PHA biopolymer as a P (HB-*co*-HV) copolymer.

The ^1^H NMR spectrum (Figure 7) showed a clear peak at about 5.25 ppm that corresponds to the proton on the chiral carbon close to the ester oxygen in the polymer backbone. This is characteristic of short-chain-length PHA (scl-PHA), like PHB. The presence of this peak is the strongest evidence for the dominance of 3HB units in the polymer structure. The methine peak at 5.25 ppm was broad, which may indicate a small amount of 3-hydroxyvalerate (3HV) as a copolymer. The methylene (-CH_2_-) protons next to the carbonyl carbon make up the group of peaks between ~2.5 and 2.2 ppm in the spectrum, with minor peaks at 2.26, 2.33, and 2.48 ppm. This region corresponds to the hydrogens of the methylene group in the β position relative to the carbonyl group. The splitting pattern of this peak (multiplet) shows a more complex chemical environment and the possibility of different sequences (such as 3HB-3HV), but it still falls within the main range of scl-PHAs. The protons of the methyl (-CH_3_) side groups handle the broad signals between about 1.6 and 1.2 ppm. This region corresponds to the hydrogens of the terminal methyl group in the side chain of the 3HB unit. This range is pretty normal for scl-PHAs. This pattern fits well with PHB and is in line with earlier ^1^H NMR results for PHAs produced by Bacillus species [65,84,85]. The methyl region at 0.83 ppm also shows the presence of 3HV in the compound’s structure.

The ^1^H NMR spectra clearly indicate an HB-dominant copolymer. They also show that the material produced in the long-term semi-continuous fermentation was a PHA made mostly of 3-hydroxybutyrate (3HB) units and some 3-hydroxyvalerate (3HV) units. The structure remained intact despite the extended cultivation time and variable oxygen conditions, demonstrating structural stability comparable to previously reported Bacillus-derived PHAs [43,51]. Therefore, based on the NMR results, the polymer is a P (HB-*co*-HV) copolymer rather than a homopolymer.

#### 3.8.3. Differential Scanning Calorimetry (DSC)

**Figure 8:**
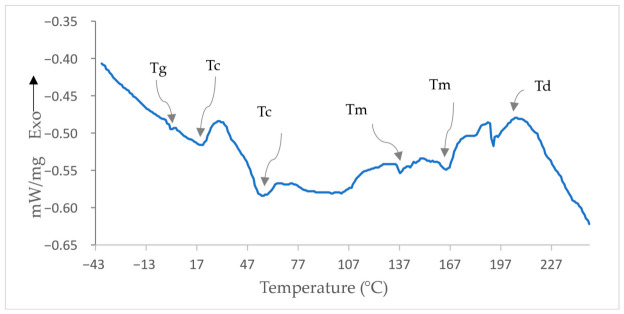
Differential scanning calorimetry (DSC) thermogram of the extracted polymer.

We used differential scanning calorimetry (DSC) to analyze the thermal properties of the isolated PHA biopolymer. Figure 8 shows the thermogram with the glass transition (Tg) area at 1.45 °C. This was followed by two endothermic shoulders that showed recrystallization (Tc) at 20.45 °C and 55.45 °C. After that, two endothermic peaks were found at 165.45 °C and 137.45 °C. These peaks show the melting point (Tm), which is normal for PHB copolymers. It was found that the polymer degradation temperature (Td) was 212.45 °C. Our results are in accordance with Don et al. [86], who reported the Tg of copolymers (3HB-co-3HV) synthesized by *Haloferax mediterranei* as 1.41 °C at a heating rate of 10 °C/min, while the Tm varied between 133 and 145 °C. The glass transition temperature is important for stability at low temperatures and varies from 4 to −44 °C as the polymer subcontents change [87]. The glass transition is influenced by the size of the side group. A larger alkyl chain size in the side group causes steric hindrance, leading to a lower Tg, mainly owing to an increase in the free volume [88]. Tg depends on the percentage of 3HV in the copolymer composition and the heating rate. The higher percentage of 3HV transfers the Tg temperature to lower temperatures [89]. Based on our Tg temperature (1.45 °C), the percentage of the 3HV in our copolymer must be low.

Different Tc values show side-chain crystallization owing to different monomer contents in the copolymer. A secondary crystallization peak appeared at 54.95 °C, consistent with recrystallization under a controlled thermal history and in line with values typically reported for PHB [90].

PHA copolymers have different monomer compositions and contents, which confer unique physical and thermal properties [91,92]. Thermal property measurement is important for the future use of PHA polymers. The melting point of a polymer should be substantially lower than its degradation temperature. The polymers in this study exhibited melting temperatures ranging from 137.45 °C to 165.45 °C. These two melting points were lower than the melting point of pure P (3HB), which was reported at 175 °C. Similarly, in *B. megaterium* ATCC 14945, two endothermic peaks at 133.15 and 145.42 °C were reported [54,93]. The presence of two melting peaks (Tm) is commonly attributed either to the presence of two isomeric forms (α and β) or to the incorporation of HV or HHP copolymers, which have different thermal stabilities. The smaller, imperfect crystals melt at a lower temperature (137.45 °C). As the temperature increases, the chains can rearrange and form more perfect and stable crystals, which then melt at a higher temperature (165.45 °C) [86]. The degradation temperature of our polymer was 212.45 °C, which was greater than its melting temperature.

#### 3.8.4. Thermal Stability

**Figure 9:**
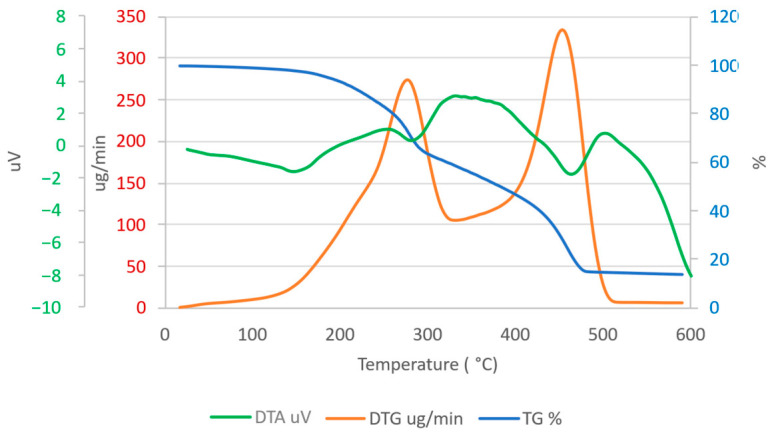
Thermogravimetric analysis (TG), derivative thermogravimetric (DTG), and differential thermal analysis (DTA) thermograms of the extracted PHA.

The thermal stability and degradation characteristics of the polymer were measured with TG/DTG/DTA. The TG, DTG, and DTA curves showed a multi-step thermal degradation process, consistent with the previously recorded thermal behavior of microbially generated PHAs (Figure 9) [94,95]. A slight weight loss below 150 °C was observed, attributed to the evaporation of kept moisture and residual volatile chemicals from the extraction process, a typical feature of bio-based polymers such as PHA [59,96,97]. The TG curve shows that the polymer remained stable at temperatures up to approximately 200 °C. The main degradation started at a temperature of 210–220 °C (T_onset_), and the fastest breakdown happened at a temperature of 275–280 °C (T_max_), as shown by a clear DTG peak. This first step has to do with breaking the ester bonds in the polymer, which is the main way that PHAs break down when they are heated [96,98]. Between 300 and 400 °C, there was a steady loss in weight, along with a wide DTG and DTA region. These results show that multiple structural phases of the polymer were breaking down, which is a common pattern for PHAs with moderate crystallinity [55]. Between 420 and 482 °C, a final and more noticeable mass loss occurred. Strong thermal stability may result from random co-monomer chain lengths, which can cause high side-chain crystallization, which provides the polymer with thermal stability [99,100,101]. The weight remained the same after 482 °C, and 15–20% of the mass remained after 500 °C. This residue is prevalent and likely originates from inorganic salts in the growth medium or the generation of stable carbonaceous material, especially in the absence of rigorous purification [79]. The DTG curve displayed two weight loss rates, which may reflect the evaporation rates of two different monomers [102]. We can conclude that PHAs generated by *Bacillus cereus* from fishmeal wastewater comprise multiple monomer types. The T_onset_ and T_max_ values obtained in this investigation closely align with those documented for poly(3-hydroxybutyrate) (PHB). Our findings show that the biopolymer primarily comprises PHB [94,103]. In conclusion, the thermal study validated that our biopolymer exhibited sufficient stability at processing temperatures nearing 200 °C, and its degradation profile aligned with its expected chemical structure, hence endorsing its prospective use in scenarios causing moderate thermal resistance.

#### 3.8.5. Morphological Characterization (SEM Analysis)

**Figure 10:**
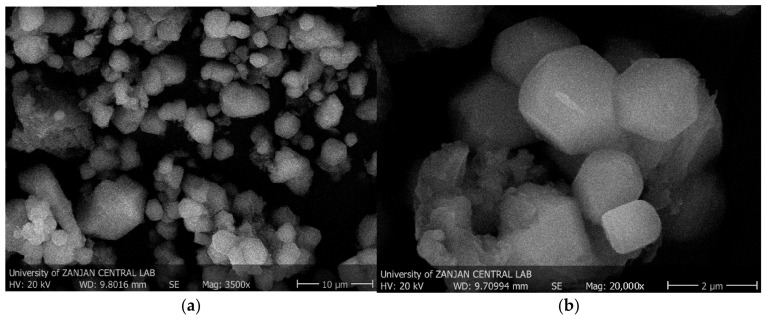
SEM micrographs of the extracted PHA biopolymer: 3500× magnification (**a**) and 20,000× magnification (**b**).

We examined the surface morphology of the extracted PHA using scanning electron microscopy (SEM). Heterogeneous particulate structure, dispersed within the micrometer scale range, was investigated (Figure 10).

The particles were not evenly spread and had a strong tendency to stick together. This tendency is due to the strong interactions between the polymer chains and the drying stage after extraction, which often causes the particles to stick together. Similar agglomerated morphologies have been widely described for bacterially generated PHAs and are distinctive features of these materials [79,94].

As shown in Figure 10, the single particles were mostly circular but not perfectly circular. They ranged in size from less than a micron to several micrometers in diameter. The surfaces of the particles were mostly smooth and did not have any large fissures or fibrous damage. This shows that our extraction and purification techniques did not significantly impair the polymer structure. Poly(3-hydroxybutyrate) (PHB) and other PHAs with simple compositions often have smooth, round shapes [83,104]. The morphology of the particles matched the biological source of PHAs, as bacteria produce and store these polymers as tiny granules. These granules maintain most of their original particle shape after being removed. The tendency of these particles to clump together on a micrometer scale can change the flow and behavior of the material during processing. This shows how important it is to examine the shape of a material to determine if it can be used in real life [59,94].

Based on the SEM results, the isolated PHA was composed of irregular micrometer-sized particles that stuck together. The morphological characteristics identified in this study align with those documented in the literature for PHAs, especially for materials directly associated with PHB.

#### 3.8.6. X-Ray Diffraction (XRD) Analysis

**Figure 11:**
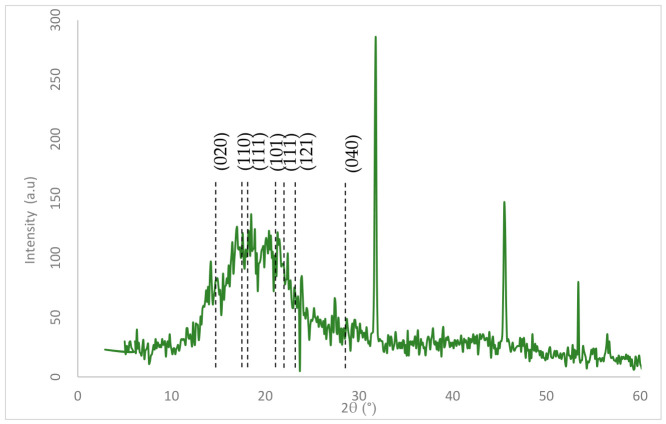
X-ray diffraction (XRD) pattern of the sample in the 2θ range of 5–60°.

The synthesized biopolymer’s X-ray diffraction (XRD) pattern was measured in the 2θ range of 5–60° (Figure 11). The XRD pattern exhibited a broad peak region between 2θ ≈ 13° to 30°, featuring a central maximum at approximately 2θ ≈ 20.1°, along with several peaks around it. This pattern is characteristic of a semicrystalline polymer. The crystallographic planes were assigned to the XRD pattern. The analysis results revealed the presence of several diffraction peaks. The peaks appeared at 13.7°, 16.8°, and 20.1° angles, corresponding to the (020), (110), and (100) crystal planes in the orthorhombic lattice of PHB. Four additional peaks were observed at angles of 17.6°, 21.3°, 22.4°, and 27.4°, which can be attributed to the (111), (111), (121), and (040) planes [105,106].

For microbial biopolymers like poly(3-hydroxybutyrate) (PHB) and PHBV, recognizable crystalline peaks usually appear around 2θ ≈ 13.5°, 16.8°, and 20.1° [55]; these were observed at 13.7°, 16.8°, and 20.1° in the present sample. Crystallinity (Xc) was calculated as 60.17%. This confirms the semicrystalline structure of biopolymers. This is in agreement with another study on 3HB-co-3HV, which reported 62.3% crystallinity [107].

## 4. Conclusions

This study examined *Bacillus cereus* as a prospective producer of polyhydroxyalkanoate (PHA) using fish-processing plant effluent as an accessible, straightforward, and economical carbon source while assessing its capacity to generate biopolymer under diverse cultivation conditions. The findings show that this bacterium can produce PHA with significant yield, and the cultivation conditions are crucial in influencing the level of polymer accumulation and its monomeric composition. We could extract and purify the polymer, and the tests showed the product was a copolymer. FTIR analysis confirmed the characteristic functional groups of PHA, and XRD analysis showed that the polymer was semicrystalline. Thermal analyses conducted via DSC and TGA showed that the synthesized polymer displays unique melting and crystallization characteristics, along with adequate thermal stability. The agreement between the structural and thermal results and the identified composition suggests that the copolymeric structure plays a role in determining its physical properties. The results suggest that *B. cereus* is a promising microbial source for generating semicrystalline copolymeric (3-PHB-*co*-3-PHV) with favorable thermal properties. These results may pave the way for the development of more cost-effective biological processes and the production of biodegradable biopolymers with industrial and engineering applications.

## Figures and Tables

**Table 1:** Physicochemical characteristics of raw fishmeal wastewater.

Parameter	Value
pH	6.67
Total solids (TS)	144,533.3
Total suspended solids (TSS)	80,244
Chemical oxygen demand (COD)	112,000
Total organic carbon (TOC)	37,316.9
Total Kjeldahl nitrogen (TKN)	462
Total ammonia nitrogen (TAN)	124.05

All values, except pH, are expressed in mg/L.

**Table 2:** Effect of wastewater dilution on bacterial growth and PHA production.

Dilution (%)	CDM (g/L)	PHA Content (%)	PHA Yield (g/L)
25%	0.347 ± 0.13 b	8.87 ± 3.45 a	0.03 ± 0.01 a
50%	0.426 ± 0.165 b	6.69 ± 3.54 a	0.027 ± 0.01 a
75%	0.726 ± 0.051 a	7.04 ± 2.07 a	0.052 ± 0.02 a
100%	0.433 ± 0.064 b	5.81 ± 3.27 b	0.024 ± 0.01 a

Data are shown as mean ± SD. Letters “a” and “b” show significant differences in each column (*p* < 0.05).

**Table 3:** Effect of fermented substrate on bacterial growth and PHA production.

Treatment	CDM (g/L)	PHA (g/L)	PHA Content (%)
50% Unfermented	0.424 ± 0.165 a	0.0172 ± 0.013 a	6.69 ± 3.54 c
50% Fermented	1.022 ± 0.281 c	0.375 ± 0.113 d	31.87 ± 5.76 a
100% Unfermented	0.433 ± 0.064 a	0.0243 ± 0.013 a	5.81 ± 3.27 c
100% Fermented	0.970 ± 0.217 b	0.249 ± 0.069 c	25.64 ± 3.97 b

Data are shown as mean ± SD. Same letter: no significant difference. Different letters: significant difference (*p* < 0.05).

## Data Availability

The data generated during this study are included in this published article.

## Abbreviations

The following abbreviations are used in this manuscript:

PHA	Polyhydroxyalkanoate
FPW	Fish-processing wastewater
COD	Chemical oxygen demand
CDW	Cell dry weight
CDM	Cell dry mass
FTIR	Fourier-transform infrared spectroscopy
NMR	Nuclear magnetic resonance
DSC	Differential scanning calorimetry
TGA	Thermogravimetric analysis
SEM	Scanning electron microscopy
XRD	X-ray diffraction
TS	Total solids
TSS	Total suspended solids
TKN	Total Kjeldahl nitrogen
TAN	Total ammonia nitrogen
TOC	Total organic carbon
VFAs	Volatile fatty acids
EPS	Extracellular polymeric substances
LCFAs	Long-chain fatty acids
PHB	Poly(3-hydroxybutyrate)
3-HD	3-hydroxyhexadecanoic acid
